# Digital versus screen film mammography: a clinical comparison

**DOI:** 10.2349/biij.4.4.e31

**Published:** 2008-10-01

**Authors:** Y Faridah

**Affiliations:** 1 Department of Biomedical Imaging, Faculty of Medicine, University of Malaya, Kuala Lumpur, Malaysia

## INTRODUCTION

Breast carcinoma is the most common malignancy among women in developed countries, and its incidence is on the rise in developing countries. In Malaysia, it accounts for about 30% of newly-diagnosed female cancers [1]. Imaging of the breast can be traced to its earliest origins in 1913 when a surgeon, Albert Salomon, used radiography images of mastectomy specimens to demonstrate the spread of breast carcinoma to the axillary lymph nodes. However it was not until 1960 that the widespread use of mammography became possible. This was attributed to Robert L. Egan who described a mammography technique that was reproducible. The first x-ray unit dedicated to breast imaging was available by 1965. By the 1970s, mammography as a screening device became standard practice [2]. This was because mammography by then had been proven to be the most effective method of detecting early breast carcinoma. The use of mammography in screening of breast carcinoma has been found to significantly reduce the mortality of this disease [3].

The transfer of imaging to the digital format began two decades ago with the introduction of digital radiography. By natural progression, other imaging modalities then adopted the digital technology. The transition from conventional mammography to its digital counterpart, however, was delayed due to the difficulty of producing a full-field digital detector [4].

The first full-field digital mammography unit was approved for sale by the Food and Drug Administration in 2000 [5]. Since then numerous hospitals and medical centres worldwide have installed digital mammography systems.

## PERFORMANCE OF DIGITAL MAMMOGRAPHY (DM)

With any new technology, there is a need to compare its performance with the known gold standard. Screen-film mammography (SFM) is the gold standard for breast cancer detection. The SFM technology had been perfected over the years and mammography unit personnel the world over had been well trained in this technique. Its quality protocols for breast cancer detection and screening are also well established. SFM also has a high spatial resolution well suited for detection of microcalcifications, one of the signs of early breast carcinoma.

### Detection of carcinoma

One of the drawbacks of SFM is its contrast resolution. The breast is a difficult organ to image as it consists of tissues of contrasting densities; glandular tissue interspersed with fat. The amount of glandular tissue varies in different women of different ages, ranging from dense (in which 75% or more of the breast is occupied by glandular tissue) to fatty. It has been found that women with dense breasts have a four to six times higher risk of breast cancer compared to women with little or no glandular tissue. This is postulated to be due to the masking of existing lesions by the overlying breast tissue [6]. Therefore the sensitivity of mammography in detecting carcinoma in dense breasts is limited; a 62.9% reduction in sensitivity in dense breasts as compared to 87.0% in breasts with fatty involution [7].

When comparing DM to SFM, it was found that the overall diagnostic accuracy of both technologies in detecting breast cancer detection was similar [8, 9]. However, the DM was found to be more accurate in women under 50, women with dense breasts and in premenopausal and perimenopausal women [8]. This is likely to be due to the wide dynamic range of DM that offers an advantage over SFM. DM is able to capture areas of contrasting densities and display these regions without compromising the contrast resolution very much.

**Figure 1:**
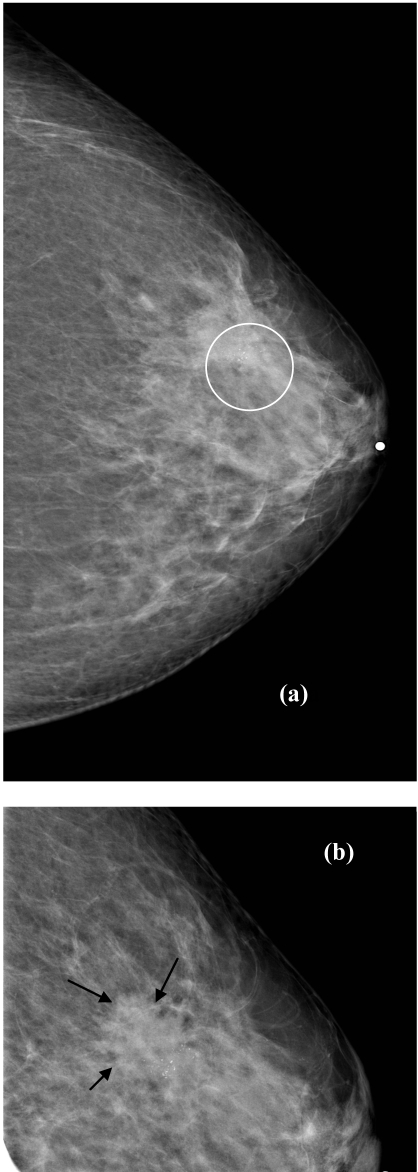
(a) Area of microcalcification seen on the left craniocaudal (CC) view (white circle). (b) Calcifications were better appreciated on compression - magnification view. An associated ill-defined mass was also appreciated (arrows).

As mentioned before, SFM boasts a high spatial resolution of approximately 16 linepairs per mm which enables detection of fine structures such as microcalcification. The spatial resolution of DM, however, is limited by pixel size. Despite this limitation, it has been found that the detection of microcalcifications on DM is equal to, if not better than, that of SFM [10, 11] (Figure 1). In a study by Fischer U et al., DM showed more calcification compared to SFM, having a higher sensitivity and reliability in characterising microcalcification. This is due to the increased contrast resolution of DM which enhances its ability to visualise small high-contrast structures such as microcalcification [10].

The advancement of digital imaging now allows new techniques of breast cancer detection. Two such techniques that show promise are contrast-enhanced mammography and tomosynthesis. In contrast-enhanced mammography, a contrast agent, usually iodine–based, is administered following an unenhanced image acquisition. As post-contrasted images are acquired (either using temporal or dual energy technique), these images are subtracted exposing the pathology in the breast without breast tissue superimposition. In tomosynthesis, the use of a stationary detector with a moving x-ray source results in images of the breasts obtained from different angles. Structures within the breasts are then shifted against each other, again giving rise to images with less breast tissue superimposition [12].

### Image manipulation and post-processing

Another advantage of DM is the ability to manipulate the digital information after exposure has been made. With SFM, an image that has been under- or over-exposed will lose its diagnostic quality and would need to be repeated. With DM the repeat rate is found to be low [13].

**Figure 2:**
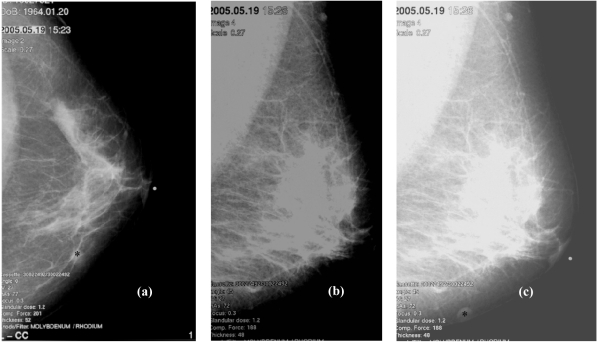
(a) A rounded nodule (*) in seen at the inner quadrant in the left craniocaudal (CC) view. (b) The nodule was not seen on the left mediolateral oblique (MLO) view. (c) The nodule (*) was appreciated to be within the subcutaneous tissue following contrast manipulation on MLO and was diagnosed as a sebaceous cyst.

**Figure 3:**
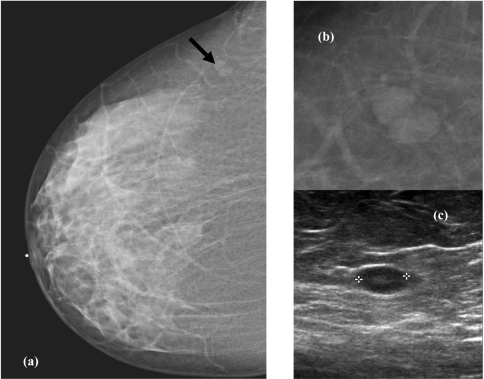
(a) Nodule at right outer quadrant on right CC view (black arrow). (b) Magnification of the nodule shows a rim of lucent halo suggestive of a benign nodule. (c) Ultrasound confirms a benign intramammary lymphnode with a central fatty hilum.

**Figure 4:**
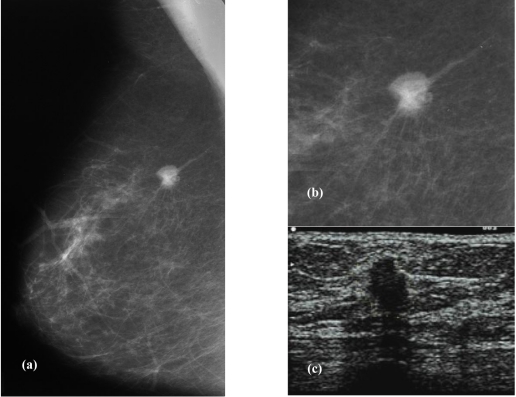
(a) A nodule seen on the right MLO view (arrow). (b) Magnification showed the same nodule to have a spiculated margin suspicious of malignancy. (c) Ultrasound confirms the presence of an invasive ductal carcinoma (biopsy proven) showing a hypoechoiec nodule with a ‘taller than wide’ appearance.

**Figure 5:**
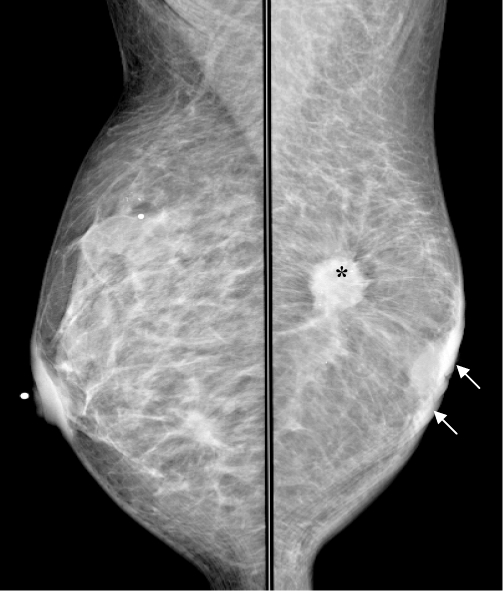
An invasive ductal carcinoma (*) giving a stellate appearance in the left breast on MLO view. There is associated thickening of the skin (white arrows) well appreciated on this digital mammogram.

DM allows for manipulation of the image contrast (Figure 2) and the ability to zoom and magnify (Figures 3 and 4). The overall image also delineates soft tissue better, especially the subcutaneous skin, an area that was not well seen on SFM (Figure 5). It is important to realise that radiologists need to report off the workstation monitors to fully utilise the ability to manipulate images in DM. This will necessitate training of radiologists to familiarise themselves from hard-copy to soft-copy reporting. Another important point to remember is that no amount of image manipulation could compensate for a mammogram that had been taken using unsuitable exposure parameters. A good mammogram, be it using SFM or DM, performed by conscientious radiographers and skilled interpretation by radiologists will yield the optimum results.

In a recent survey by Haygood et al., it was found that magnification (72.4% of respondent) is the main tool that is often used by radiologists while reading a digital mammography examination [14]. It has been suggested that the use of these computer-based tools such as zoom and magnify may replace or minimise dedicated compression-magnification view. To date, however, this has not been proven as computer-based magnification of images contains less rather than more additional information and hence would not be able to theoretically replace dedicated compression-magnification views [15].

### Workflow process

Converting from an SFM to a DM system improves the throughput of mammography cases mainly due to a more efficient workflow [16]. There is a 45% reduction in the time taken to perform examinations and process images using DM when compared to SFM [13]. The majority of time saved is in the abolishment of film processing time [16]. This aspect also proves useful in interventional breast procedures such as hook-wire localisation, as images can be viewed immediately on the console without the extra time taken to process films in between each step of the procedure [15]. Furthermore, hard-copy images of DM is of a more consistent quality compared to SFM, as the conventional method of film processing in SFM resulted in variable images sometimes fraught with artefacts.

The time taken to interpret soft-copy DM images has been found to be longer compared with SFM [14, 16]. This is mainly attributed to the time taken to manipulate the image by using available tools such as zoom and magnification on the workstation. However there is no significant difference when comparing the speed and accuracy of interpretating soft-copy versus printed-film digital mammography [17]. Difficulty also arises when comparing a current soft-copy digital examination with a previous SFM examination, as direct side-by-side comparison is almost impossible. Furthermore, illumination from a viewing box placed next to a workstation may interfere with the image display on the workstation [15]. As no consensus has been reached regarding this problem, it will be up to the individual to decide on the best method to overcome it.

### Image archival, storage and retrieval

In DM, the images are stored in digital format such as on magnetic optical discs or compact discs. This will considerably reduce the demand for storage space when compared to SFM. The quality of the images stored is also preserved as hard-copy images do not degrade due to poor storage conditions. Transfer of images from remote locations is also possible, opening the door to teleradiology. With the introduction of PACS (Picture Archiving and Communication System), the demand for digital imaging increases and the ability of imaging departments to go film-less is realised, further improving the standards of healthcare.

### Cost effectiveness

One of the prohibitive factors to the advancement of digital mammography is its cost, estimated to be 1.5 to 4 times higher than SFM [8]. In a recent study by Tosteson A et al, it was found that while it is beneficial to screen for breast carcinoma in younger women (especially those with dense breasts) using digital mammography, the same could not be said for older women (especially those with non-dense breasts). In the older women, screen-film mammography may offer better detection of breast carcinoma. Overall it was found that using all-digital mammography in screening for breast carcinoma is not cost-effective and that age-targeted digital mammography screening is the most efficient approach when considering healthcare provisions [18]. Therefore a balance has to be achieved between desire and reality (the actual need).

## CONCLUSION

Digital mammography has been shown to be as good as SFM in detecting breast carcinoma, and even performs better in women with dense breasts. DM improves the mammography workflow and therefore increases the throughput. It does entail a high cost, which is the main prohibitive factor in its advancement. With robust technological advancement, however, drawbacks could eventually be overcome, opening the door for DM to replace SFM as the gold standard for breast cancer detection.
